# Linear and conformational epitopes of vicilin-buried peptides as a model for improved nut allergy diagnostics

**DOI:** 10.3389/falgy.2025.1648262

**Published:** 2025-09-22

**Authors:** Lauren T. Swientoniewski, Ian M. Rambo, Jacqueline B. Nesbit, Hsiaopo Cheng, Stephen A. Y. Gipson, Stacie M. Jones, Dieu T. Doan, Stephen C. Dreskin, S. Shahzad Mustafa, Scott A. Smith, Michael D. Kulis, Adam R. Rivers, Alexander C. Y. Foo, Geoffrey A. Mueller, Soheila J. Maleki

**Affiliations:** 1 Food Processing and Sensory Quality Research Unit, United States Department of Agriculture, Agriculture Research Service, New Orleans, LA, United States; 2 Department of Biology, Delgado Community College, New Orleans, LA, United States; 3 Division of Allergy and Immunology, Department of Pediatrics, Arkansas Children's Hospital, University of Arkansas for Medical Sciences, Little Rock, AR, United States; 4 School of Medicine, University of Colorado Denver, Denver, CO, United States; 5 Rochester Regional Health, University of Rochester School of Medicine and Dentistry, Rochester, NY, United States; 6 Department of Pathology, Microbiology and Immunology, Vanderbilt University Medical Center, Nashville, TN, United States; 7 Department of Pediatrics, University of North Carolina School of Medicine, Children’s Research Institute, Chapel Hill, NC, United States; 8 Genomics and Bioinformatics Research Unit, United States Department of Agriculture, Agriculture Research Service, Gainesville, FL, United States; 9 Division of Intramural Research, National Institute of Environmental Health Sciences, Durham, NC, United States

**Keywords:** cross-reactivity, peanut allergy, tree nut allergy, IgE, vicilin (7S), epitopes, vicilin leader sequence, vicilin-buried peptide (VBP)

## Abstract

**Introduction:**

Individuals allergic to peanuts (PN) may show IgE cross-reactivity to tree nuts, especially walnuts (WN), which often complicates diagnosis. Vicilin-buried peptides (VBPs), short segments within the N-terminal vicilin leader sequence (LS), contribute to cross-reactivity due to their ubiquitous, highly conserved and stable *α*-hairpin structures. The binding patterns of cross-reactive IgE to linear and conformational epitopes of PN and WN LSs and constituent VBPs may serve as a model for understanding clinically symptomatic cross-reactivity.

**Methods:**

Serum samples (*n* = 30) from primarily oral food challenge-positive individuals with PN allergy (PNA, 33%), WN allergy (WNA, 47%), and PN and WN allergies (PWA, 20%) were collected. These sera and a monoclonal IgE antibody (6D12) were examined for IgE binding with microarrays of overlapping peptides from native Ara h 1 LS [AH1LS, Ara h 1.0101 (26–84)] and recombinant Jug r 2 LS [JR2LS, Jug r 2.0101 (1–173)] and via direct and competitive inhibition ELISA with intact LSs and constituent VBPs from PN (AH1.1) and WN (JR2.1, JR2.2, JR2.3). A mixed model analysis assessed the contribution of IgE binding patterns to VBPs in relation to PNA, WNA, or PWA status.

**Results:**

All three intact WN VBPs bound IgE at similar frequencies, with individual sera showing varying preferences for specific VBPs. AH1.1 was less recognized by WNA individuals but more frequently recognized by PNA and PWA subjects. WN VBPs were recognized by PNA sera samples at rates comparable to AH1.1. Our data indicates that each VBP can bind to one IgE molecule with high affinity. In a competitive inhibition ELISA, combining VBP competitors did not enhance inhibition compared to the dominant VBP, suggesting that both high- and low-affinity VBPs compete for the same monoclonal IgE in serum. This observation was mimicked by 6D12, a monoclonal IgE against JR2.1.

**Discussion:**

Cross-reactivity among VBPs most likely arises from monoclonal IgE binding to α-hairpin structures and their overlapping linear amino acid sequences. The combination of linear and conformational IgE binding patterns enabled us to differentiate between the WNA, PNA, and PWA groups in this study and may assist us in using AH1LS and JR2LS to distinguish PN and WN allergies in the future.

## Graphical Abstract

**Figure:**
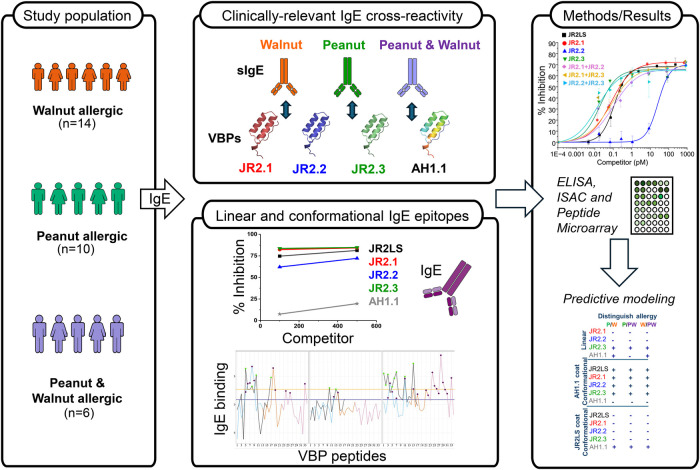

## 1 Introduction

Peanut and tree nut allergies, such as walnuts, pecans, cashews, and pistachios, co-exist in ∼30% of food-allergic individuals (1, 2). The cause of co-existing allergies to peanuts and tree nuts has been extensively studied and is frequently attributed to cross-reactive epitopes among individual food allergens (3).

Food allergy cross-reactivity occurs when an individual's serum IgE against an allergenic protein fails to discriminate between the original sensitizing allergen vs. another protein. These other proteins are often structurally and sequentially similar to the sensitizing allergen and result in IgE binding that may elicit an allergic response or may be clinically irrelevant (4–7). Therefore, it is critical to elucidate the nature of the antibody-allergen binding and identify the IgE epitopes and their corresponding properties that facilitate cross-reactivity. Almost 60% of major plant food allergens are categorized in four protein families: prolamin, cupin, profilin, and the Bet v 1 superfamily (8). Cupins are a family of seed storage proteins that include vicilins that are found in plant seeds, including peanuts and tree nuts. Vicilins (7S globulin) contain three conserved regions: a signal peptide (∼25 amino acids long), which acts as an “address label” and directs newly synthesized vicilins to the protein bodies within the seed, an N-terminal cysteine-rich leader sequence (LS), originally thought to be a part of the vicilin and used as a nitrogen source for seed development (9), and the mature vicilin domain (i.e., Ara h 1 and Jug r 2). The focus of this study is what we refer to as the LS, which contains a ubiquitous family of peptides called vicilin-buried peptides (VBPs), characterized by a common α-hairpin fold (α-hairpinin motif) mediated by two disulfide bonds between conserved CxxxC motifs (8, 10) and thought to play a role in pathogen resistance. Originally the peanut vicilin LS, referred to as Ara h 1 LS (AH1.1) here, was considered part of the mature vicilin and reported to contain three of the immunodominant linear epitopes of Ara h 1 (11). It was later understood that the N-terminal region is most often a separate domain that is cleaved from the vicilin (9), and it has since been identified in the seed (12, 13). More recently, studies have shown that VBPs in peanut, cashew, pistachio, and walnut are IgE-reactive, introducing these sequences as a new family of allergens (14, 15). Ara h 1 and Jug r 2 LS were submitted to WHO IUIS Nomenclature committee and officially named Ara h 1.10101 (26–84) and Jug r 2.0101 (1–173). Additionally, IgE epitopes in the vicilin LS of Jug r 2 were previously identified and recognized based on a combination of sequence alignment and structure similarities to an immunodominant epitope in Ara h 2, the most recognized and potent peanut allergen (16–18).

**Figure 1:**
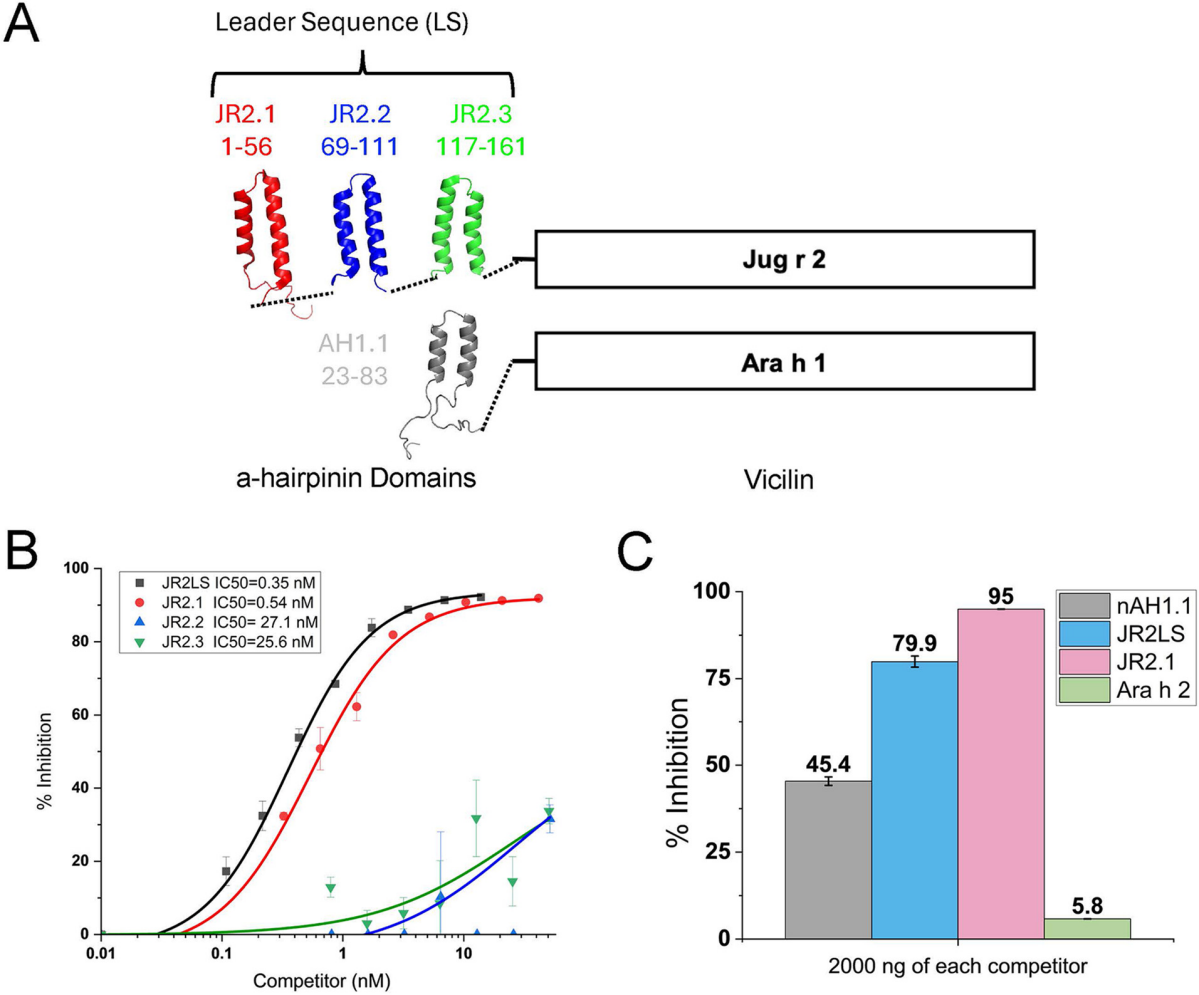
Competitive inhibition ELISA for binding to 6D12 monoclonal antibody. **(A)** Vicilin-buried peptides (VBPs) are found at the N-terminus in seed-storage vicilin proteins. The leader sequence of the walnut vicilin Jug r 2 contains three identified VBP motifs (JR2.1, JR2.2, JR2.3) whereas the peanut vicilin Ara h 1 contains only one (AH1.1). **(B)** The percentage of inhibition (*y*-axis) of binding to JR2LS by increasing amount of competitor (indicated in the figure inset, including IC_50_ values for each curve) is shown on the *x*-axis. The R^2^ for the logistic fitting of the percent inhibition data for JR2LS and JR2.1 was 0.9926 and 0.99622 respectively. **(C)** Inhibition of 6D12 monoclonal antibody binding to an ELISA plate coated with intact JR2LS. Percent inhibition (*y*-axis) was calculated for different competitors (figure inset) at 2000ng each.

The LS of Jug r 2.0101 (JR2LS) is comprised of multiple VBP motifs, and the three closest to the vicilin domain have been identified and are assessed here (JR2.1, JR2.2, and JR2.3). Both the intact LS and fragments thereof are found in the walnut seed (17). The LS of the peanut vicilin, Ara h 1 (AH1LS), contains a single VBP motif (AH1.1) and is therefore the same as the entire AH1LS (12) (Figure 1A). Complete three-dimensional (3D) structures of these four VBPs have been determined by NMR spectroscopy (14). IgE epitopes of these VBPs were identified with peptide microarrays, and the highest number of linear IgE epitopes were located in JR2.1 (14).

Our study seeks to address the diagnostic ambiguity caused by IgE cross-reactivity in nut allergies through three key objectives. First, we will investigate IgE cross-reactivity by utilizing known cross-reactive VBPs—small, structurally similar molecules. This involves analyzing sera from individuals with confirmed PN and WN allergies to understand the dynamics of IgE binding, specifically examining whether multiple VBP-specific IgE clones bind to different VBPs or if competition occurs between VBPs for a single monoclonal IgE. Second, we aim to analyze the linear and conformational IgE epitopes of VBPs in individuals with clinical histories of PN, WN, and PW allergies. This includes elucidating linear IgE epitopes and IgE-allergen interactions in sera from individuals confirmed to be allergic through oral food challenges (OFC+) or documented severe allergic reactions. The ISAC IgE binding profiles will validate allergic status and reveal the percentage of individuals who may be misdiagnosed with a single diagnostic tool. Finally, our research aims to enhance allergy diagnostics by demonstrating the significance of VBPs and similar structures in cross-reactivity while highlighting the critical roles of both linear and conformational epitopes in improving food allergy diagnostics. We believe these objectives will contribute valuable insights into the complexities of cross-reactivity among nuts and enhance diagnostic approaches.

## 2 Materials and methods

### 2.1 Human sera

Sera from 85 individuals with confirmed allergies (PN = 36; WN = 28; PW = 21), validated through oral food challenges or documented convincing histories, were assessed. The sera included both children and adults with known nut allergies which were assessed for binding to VBPs. Additionally, IgE epitope binding data from 10 subjects with no nut allergies were collected for comparison in the mixed model analysis. A subset of 30 sera (PN = 10; WN = 14; PW = 6) were selected for ELISA experiments due to serum availability and the ability to bind to VBPs. The screening process is further described in the ELISA section of the methods. All serum samples were obtained with informed consent and in accordance with the institutional review board regulations of the respective institutions, as well as federal policies for the protection of human subjects (Table 1). Subjects were selected based on data from food allergy serum banks and databases maintained by their institutions.

**Table 1:** Patient information^a,b^.

Subject	Age	Sex, race	WN	PN	Symptoms	Other allergy	IgE (kU/L)
1	19	F, W	✓			Other TN	
2	35	F, W	✓			Fish, sunflower, other TN	
3	Not given	Not given	✓			Other TN	
4	Not given	Not given	✓			Egg, other TN	
5	Not given	Not given	✓			Other TN	
6	Not given	Not given	✓			Other TN	
7	32	M, W	✓			None	148
8	10	F, W	✓			None	95
9	13	M, W	✓			Other TN	141
10	15	F, W	✓			Other TN	290
11	19	F, W	✓			Other TN	59
12	9	M, W	✓		MO, nLT, UA, OAS	Other TN	
13	19	M, W	✓		MO, nLT, OAS	None	
14	9	F, AA/H	✓		MO, nLT, OAS, UA, LA, OFC +	Milk, other TN	
15	20	M, W		✓	MO, nLT, OFC +	Cod, egg	>100
16	25	F, W		✓	MO, LT	Other TN	>100
17	25	F, W		✓	MO, LT, OAS	Soy	
18	6	M, W		✓	OAS, nLT	Soy	
19	9	F, W		✓	MO, OAS, UA, LA, LT, OFC +	None	
20	3	M, W		✓	MO, nLT, OFC +	None	
21	14	M, W		✓	MO, LT, OAS, UA, LA	Sesame, other TN	
22	Not given	M, W		✓	MO, nLT	None	
23	5	F, W		✓	MO, LT, OAS, LA, OFC +	Egg, other TN	
24	14	M, W		✓	MO, nLT, UA	Other TN	
25	17	M, W	✓	✓	MO, LT, OAS, OFC +	Other TN	
26	11	M, AA	✓	✓	MO, LA, nLT	Milk, sesame	
27	18	F, AA/W	✓	✓	MO, LT, OAS, UA, LA	Poppyseed, milk, other TN	
28	8	F, W	✓	✓	MO, LT, LA	Milk, egg, other TN	
29	21	F, AA/A	✓	✓		Other TN	
30	26	M, W	✓	✓		Shrimp	

a PN, peanut allergy; WN, walnut allergy; TN, tree nuts; MO, multiorgan; LT, life threatening; nLT, non-life threatening; OAS, oral allergy syndrome; OFC+, oral food challenge positive; UA, upper airway; LA, lower airway.

b In the sex, race column: M = male; F = female; W = white; AA = African-American; H = Hispanic; A = Asian. In some cases, age, sex, and race were not specified.

### 2.2 Constructs and protein purification

The intact LS and corresponding three VBPs from the English walnut (*Juglans regia*) Jug r 2 were recombinantly expressed and purified as previously described (14, 15). In brief, VBP sequences were cloned into the pDest vector and expressed in *Escherichia coli* (BL21, DE3, MilliporeSigma). Purification was performed with an immobilized glutathione column and the GST tag removed. All Jug r 2 constructs were recombinantly produced. The VBP from peanut (*Arachis hypogea*), nAH1.1, was purified by dissolving peanut flour in 100 mM sodium citrate (pH 4). The mixture was subject to ammonium sulfate precipitation at 70% saturation and cation exchange chromatography. A peak fraction containing purified nAH1.1 was identified via mass spectrometry. In this manuscript Ara h 1 LS, nAH1.1 and AH1.1 are interchangeable terms.

### 2.3 Indirect and competitive inhibition ELISA

Indirect ELISA was used to analyze 85 allergic subject sera. It is important to note that only 2 PNA subjects bound Jug r 2 VBPs and only 4 WNA subjects bound AH1.1 in the indirect ELISA, a necessary criterion for inclusion in competitive inhibition ELISA (ciELISA). Clear, high binding 96- or 384-well microplates (Corning, New York, NY, USA) were coated with 50 ng of purified JR2LS (0.05 nM in 96-well and 115 pM in 396-well) or nAH1.1 (0.15 nM in 96-well) in 0.1 M NaHCO_3_ (pH 9.6) overnight at 4°C. The plates were washed 3× with phosphate-buffered saline containing 0.05% Tween-20 (PBST), blocked with 2% dry milk in PBS for 2 h at 37°C, and washed. For indirect ELISA, diluted sera with PBS (1:2, 1:3, 1:5, or 1:10) was added to the plate and incubated for 1 h at 37°C. For ciELISA, increasing concentrations of chosen competitor(s) were incubated for 2 h, rotating, at 4°C with subject sera or 6D12, a previously described, anti-JR2.1-specific human monoclonal IgE (19) and added in triplicate to the plate for 1 h at 37°C. Plates were washed and incubated with a 1:2,000 dilution of HRP-conjugated mouse α-human IgE antibody (Southern Biotech, Birmingham, AL, USA) for 1 h at 37°C. IgE binding was detected using SureBlue^TM^ TMB Peroxidase Substrate 1-Component (SeraCare, Middlesex, UK) for 1 h at 37°C, stopping the reaction with 1M HCl. Absorbance was read at 450 nm on a microplate spectrophotometer (Tecan, Baldwin Park, CA).

### 2.4 ImmunoCAP ISAC immunoassay

ImmunoCap Immuno Solid-phase Allergen Chip (ISAC) immunoassays (Thermo-Fisher, Upsala, Sweden) were used to measure IgE binding in subject sera (Table 1) to intact, purified nut allergens as previously described (20).

### 2.5 Peptide microarray

Synthetic overlapping 15-mer linear peptides offset by 5 amino acids of Ara h 1, Jug r 2, and LSs were synthesized and spotted in triplicate onto microarray slides by JPT Peptides Technologies (Berlin, Germany). Slide preparation, analysis, and data processing were performed as previously described (20, 21).

### 2.6 Statistical analysis

Statistical analyses were conducted using R version 4.2.1 and RStudio 2022.07.0. Modified z-scores for each peptide were calculated using the IBM method, adjusting for deviations in peptide intensity using median absolute deviation (MAD). For our analyses, we used linear mixed effects models (LMMs) with the lme4 package v1.1-34, known for more accurate predictions in smaller sample sizes, to compare IgE binding to VBP regions in ciELISA and peptide microarrays across different allergic groups.

For the ciELISA data, the model assessed how IgE binding (OD_450_) varied based on different ELISA coating (C) and individual or combinations of binding competitor targets (T), (CxT) and allergic status, while accounting for participant clustering. The model was specified as OD450∼CxT * allergic status + (1 | participant id), using OD_450_ (IgE binding) as the dependent variable. This included data from 9 PNA, 14 WNA, and 6 PWA individuals. We checked for data skewness, adjusted the results to follow a Gaussian distribution, and further ensured our findings were reliable.

For peptide microarray data, we examined IgE response based on VBP regions while accounting for participant variations. The model was specified as IgE intensity∼VBP region * allergic status + (1 | participant id) + (1 | peptide number: VBP region), capturing interactions and participant clustering. Peptides included in this analysis came from the same groups as above, totaling 22 PNA, 12 WNA, 12 other allergic, and 10 controls. We transformed the data for accuracy.

Both models' performance and calculated estimated marginal means (EMMs) for pairwise comparisons were assessed, applying a correction for multiple tests to avoid false discoveries. Pairwise *p*-value plots were generated and tailored for clarity. Other statistical and graphical analyses were carried out using Origin 2023b. For further methodology details, please refer to the Supplementary Material.

## 3 Results

### 3.1 Human monoclonal IgE antibody against Jr2.1 binds specific peptide in Jr2ls

The NMR structure of AH1.1, JR2.1, JR2.2, and JR2.3 were previously solved (14) and are depicted in Figure 1A to accurately demonstrate the α-hairpin structures, the location of each VBP within the LS, and the relative structural similarity among the VBPs studied here. The mature vicilin domains in Figure 1A are labeled Jug r 2 and Ara h 1. A human monoclonal IgE antibody (6D12) was used to assess IgE binding to conformational epitopes in JR2LS and its three constituent VBPs using ciELISA (Figures 1B,C). This antibody was confirmed to have specific binding to peptide 5 (CQEYCRRQGQGQRQQ), which is located within the N-terminal helix, amino acids 21–36 of the folded JR2.1 [unpublished communications by Dr. Geoffrey Mueller (NIEHS)] (16). Our data confirms this finding in Figure 1B, where ELISA plates are coated with JR2LS and detected by the 6D12 monoclonal antibody binding in the presence of different concentrations of indicated inhibitors (*x*-axis). Based on the IC_50_ values (Figure 1B, inset), both the intact JR2LS and folded JR2.1 fragment are high affinity competitors for binding 6D12, but JR2.2 and JR2.3 are shown to be low affinity inhibitors. To determine the maximum inhibition potential by Ara h 2, previously shown to share linear IgE cross-reactivity with JR2.1, and the lower affinity inhibitors, ciELISA was performed with JR2LS coated plates detected by 6D12 antibody in the presence of saturating levels (2,000 ng) of competitors nAH1.1, JR2LS, JR2.1, and Ara h 2 (Figure 1C). At saturating levels of competitor, the percent inhibition was >75% for both JR2LS and JR2.1. The competition was significantly lower for both nAH1.1 (45.4% inhibition) and Ara h 2 (5.8% inhibition). Also, JR2.1 (a single VBP domain), showed a higher percent inhibition at a high competitor level compared to JR2LS containing three VBP domains.

### 3.2 Indirect and competitive IgE binding to the peanut VBP (nAH1.1) and all three walnut VBPs (JR2.1, JR2.2, JR2.3)

**Figure 2:**
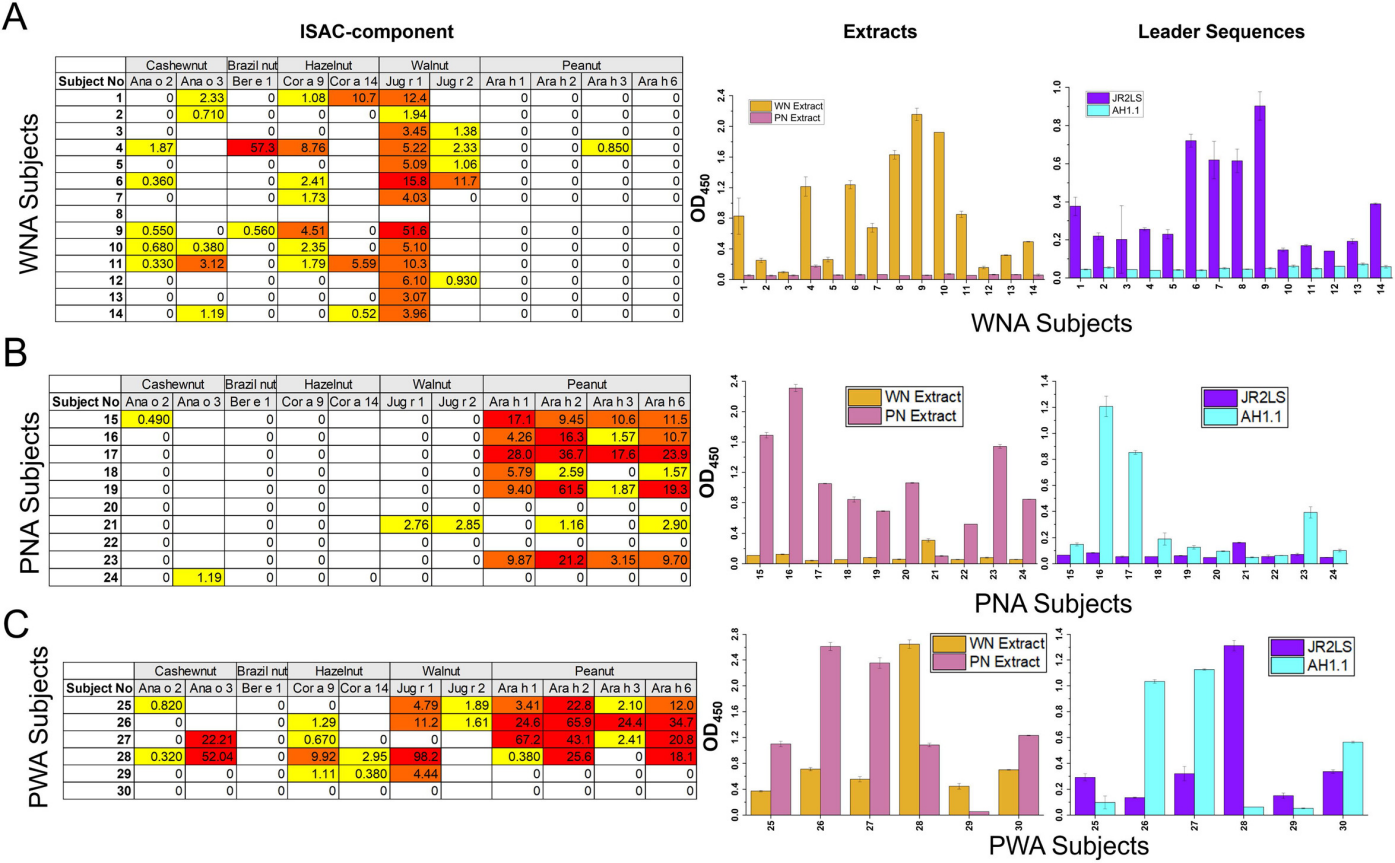
IgE binding of table I subjects to purified protein and walnut and peanut leader sequences. **(A)** ImmunoCAP ISAC immunoassay was performed on subject sera. Under the ISAC heading, the values for IgE binding to allergens in various nuts are displayed out of the 112 allergens tested in ISAC Standard Units (ISU-E). Depending on when the ISAC was performed, certain allergens (i.e., Jug r 2) were present or removed from those chip lots. IgE antibody level is indicated as <0.3 ISU (white/0), ≥0.3 to <1 ISU (yellow), ≥1 to <15 ISU (orange), or ≥15 ISU (red). Indirect ELISAs were performed with plates coated with 50 ng of either WN or PN extract (under Extracts heading, middle), or JR2LS or nAH1.1 (under Lead Sequences heading, right) using subjects in Table 1 that are WNA **(A)**, PNA **(B)**, or PWA **(C)** Absorbance values at 450 nm are shown on the *y*-axis with subject number on the *x*-axis. Error bars are SD of the mean.

AH1.1 linear IgE epitopes were originally identified by conventional methods (22), and more recent work identified IgE epitopes using peptide microarray binding by WNA, PNA, and PWA subject sera. The majority of these sequential epitopes were found in JR2.1 (14). These epitopes were localized on the ordered α-hairpinin of the VBPs, suggesting that the amino acid sequence within the conserved 3D motif was potentially mediating cross-reactivity. Here, IgE binding specificity and pattern to conformational epitopes in the VBPs were investigated by ciELISA using OFC + (or convincing history) WNA, PNA, and PWA subjects (Table 1). Subject sera IgE binding was assessed by component analysis via ISAC (Figure 2, left) to demonstrate the allergen (or component) binding preferences between WNA, PNA, and PWA study subjects (Left panels of Figures 2A–C, respectively) and to validate subject allergic status and demonstrating that a single diagnostic tool is insufficient for diagnostic accuracy. There is a clear distinction among the component binding preferences that corresponds with their diagnosed allergy. A majority of WNA subject IgE bound to purified WN allergens (Jug r 1, Jug r 2) but not to PN allergens (Ara h 1, 2, 3, and 6). The inverse can be seen with PNA subjects and a binding to both categories for PWA. There are subject sera that bound none of the expected components within their allergic group, which emphasizes the need for improved diagnostic methods.

The IgE binding levels to WN and PN extracts (Figure 2, middle) and JR2LS and AH1LS (Figure 2, right) were also measured with indirect ELISA, and the results were aligned with the ISAC data and their clinical diagnosis. Because extracts included multiple allergens, binding intensity was consistently higher to extracts vs. their corresponding LSs. An OD_450_ > 0.5 to an extract was a satisfactory predictor of subject sera binding to the corresponding LS. If sera did not bind to the whole protein extract, it was not tested for binding to the corresponding LS, as it would not be detectable. Also, if a serum did not bind to a LS corresponding to their allergic diagnosis, it indicated that the intact LS and likely the VBPs were not allergenic to that individual, and they were not included in further measurements. So, if a PNA serum did not bind to AH1.1, it was not used in AH1.1 (AH1LS) binding. In particular, serum IgE of a majority of PNA sera did not bind to JR2LS VBPs and, due to a complete lack of signal, could not be used in ciELISA with a JR2LS plate coating.

**Figure 3:**
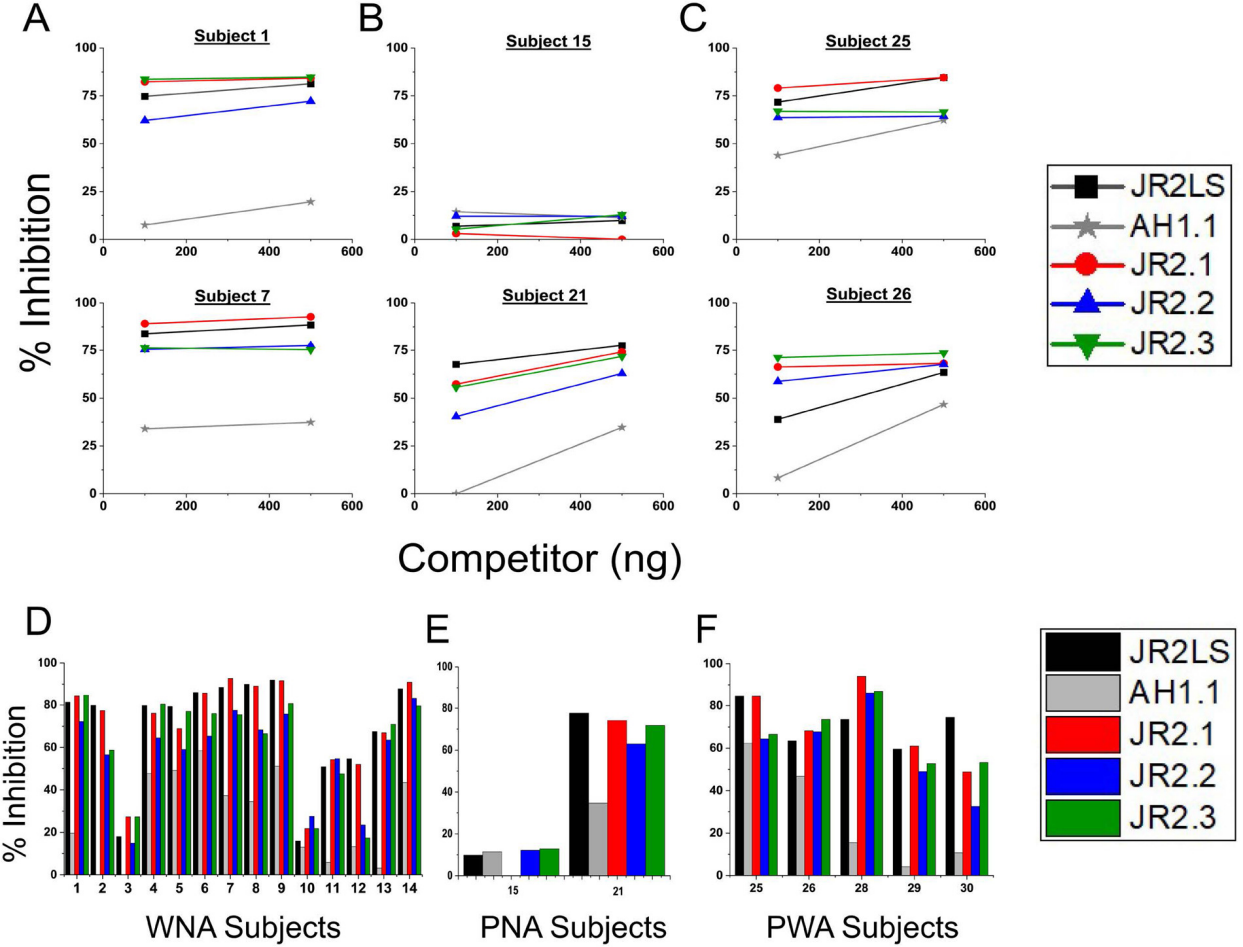
Competitive inhibition ELISA to determine IgE cross-reactivity of the peanut and walnut VBPs with intact Jug r 2 LS. Human sera with allergies to **(A)** WN **(B)** PN and **(C)** PW were used to measure the competition of four VBP fragments (indicated in the figure inset), at the two indicated levels of competitor (*x*-axis) for IgE binding to 0.05 nM of the intact Jug r 2 LS coating the wells of an ELISA plate. Two individuals from each group are shown in A-C. The level of competition by each fragment is shown as percent inhibition on the *y*-axis. The percent inhibition at 500 ng (maximum saturation) for all subjects tested with allergies to **(D)** WN **(E)** PN and **(F)** PW are shown.

Of the 85 PNA (36), WNA (28), and PWA (21) subjects screened with indirect ELISA against a combination of extracts and LSs and ISAC analysis, 30 were shown to have high enough IgE levels (and serum volume) against the LSs for use in ciELISA (Figure 2). Based on the 85 subjects that were originally screened, 20/36 (55.56%) PNA bound to AH1.1, 14/21 (66.67%) PWA bound to AH1.1, 15/28 (53.57%) WNA bound to JR2LS, and 10/21 (47.62%) PWA bound to JR2LS. For those chosen in this study, among PNA sera, 6/36 (16.67%) bound to WN extract, while only 4/36 (11.11%) bound to JR2LS (Figure 2, right), only two of which were useful for performing ciELISA, shown in Figure 3.

**Figure 4:**
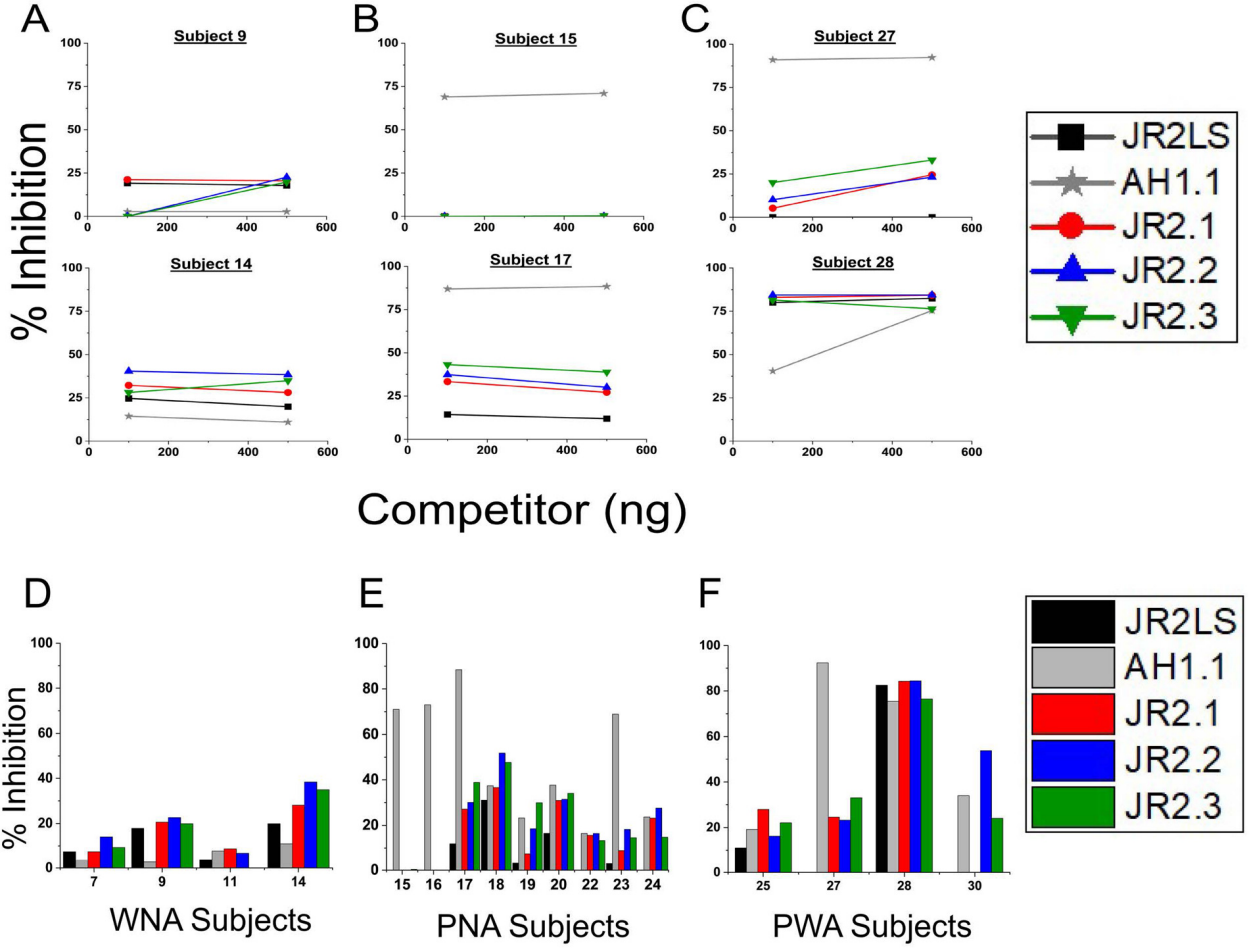
Competitive inhibition ELISA to determine IgE cross-reactivity of the peanut and walnut VBPs with intact Ara h 1 LS (nAH1.1). Human sera from individuals with allergies to **(A)** WN **(B)** PN and **(C)** PW were used to measure the competition of four VBP fragments (indicated in the figure inset) at the two indicated levels of competitor (*x*-axis) for IgE binding to intact Ara h 1 LS (0.15 nM) coating the wells of an ELISA plate. Two individuals from each group are shown in **A–C**. The level of competition by each fragment is shown as percent inhibition on the *y*-axis. The percent inhibition at 500 ng (maximum saturation) for all subjects tested with allergies to **(D)** WN **(E)** PN and **(F)** PW are shown.

To determine maximum inhibition capacity, ciELISA was used to measure the IgE binding competition by two high dose amounts (100 ng and 500 ng) of purified intact LSs and VBPs against immobilized JR2LS for all three allergic groups, indicated by panels A and D (WNA), B and E (PNA), and C and F (PWA). Representatives of ciELISA for each of the three subject groups are shown in Figure 3, in which the % inhibition at 100 ng and 500 ng for each competitor is shown on the *y*-axis. The remainder of the sera in each group can be found in Supplementary Figure S1, and the data for all subjects in each group is summarized within Figures 3D–F. While all three WN VBPs competed for IgE binding to JR2LS among subjects in each allergic group, nAH1.1 was the least competitive VBP for inhibiting binding to JR2LS regardless of subject allergic status. As previously mentioned, competitions for only 2 PNA subjects are shown in Figure 3E because in general these subjects bound very weakly if at all to JR2LS, which is a potentially useful diagnostic marker to distinguish PNA subjects. The same experimental setup was repeated with competitor LSs and VBPs inhibiting binding to immobilized nAH1.1 (Figure 4; Supplementary Figure S2). nAH1.1 was the competitor with the highest % inhibition for binding to the majority of PNA sera; however, WN VBPs exhibited higher competition with one or more of the PNA subject sera (#18, 19, 24) than nAH1.1 (Figure 4E). In ciELISA plates coated with nAH1.1 and incubated with WNA sera, the WN VBPs showed a higher percentage of inhibition than when the plates were incubated with PNA sera, which indicates that IgE in WNA sera prefers to bind to VBPs in the JR2LS over the nAH1.1. All four VBPs competed at different levels subject-to-subject for PWA for both plate coatings summarized in Figures 3F, 4F.

### 3.3 Walnut Jug r 2 VBPs compete for the same monoclonal IgE in human sera

**Figure 5:**
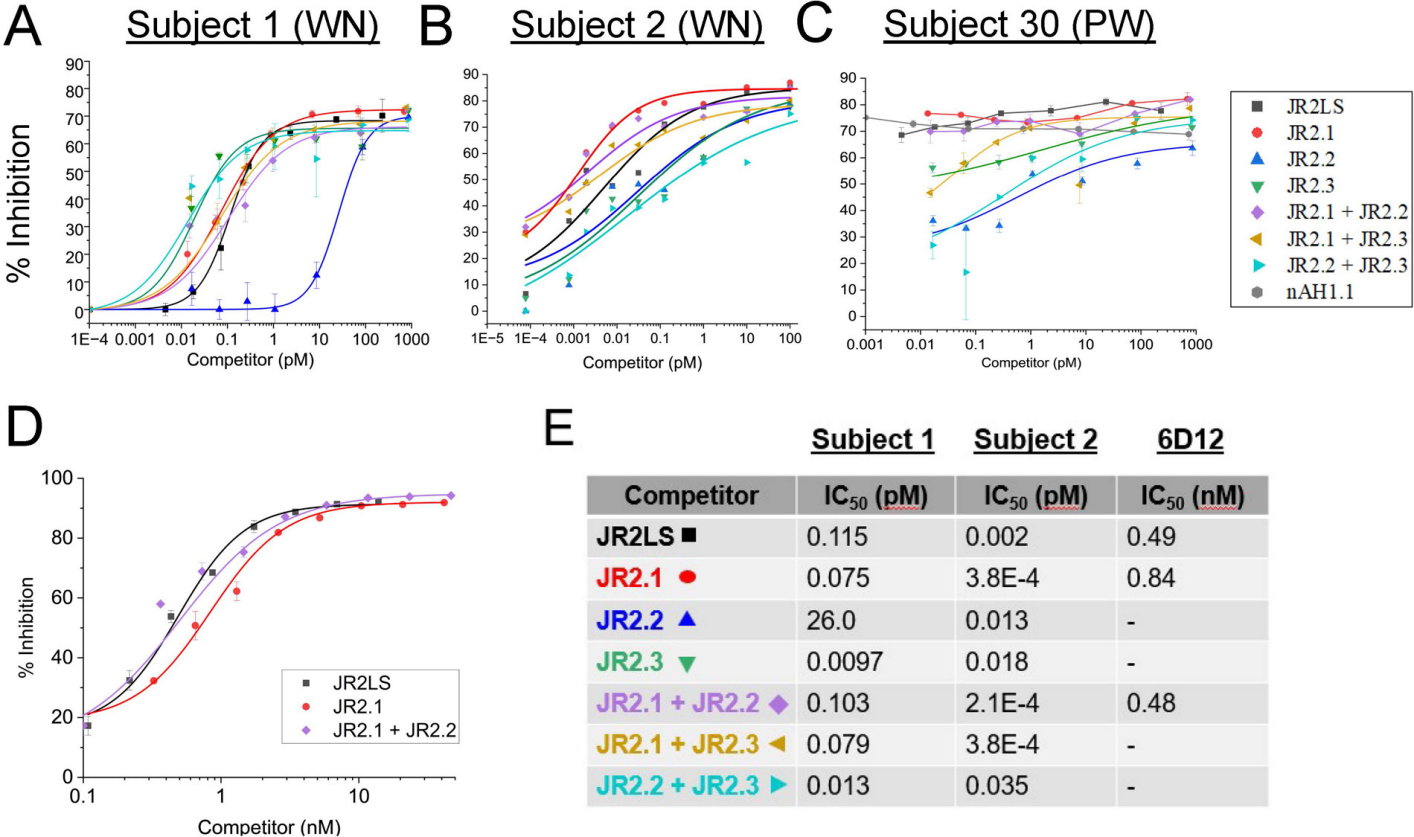
Walnut Jug r 2 VBPs compete for the same IgE molecule in sera (polyclonal) and 6D12 (monoclonal). Sera from three subjects (1, 2, and 30 in Table 1) allergic to WN **(A,B)** or PW **(C)** were used to measure the competition of four VBP fragments (indicated in the figure inset) at increasing concentrations of individual VBPs or combinations of two VBPs at equal molar concentrations (*x*-axis) for IgE binding to 115 pM of the intact JR2LS coating the wells of an ELISA plate. These subjects were chosen based on their binding levels in indirect ELISA to JR2LS and the total volume of sera available. The level of competition by each fragment and combinations thereof is shown as percent inhibition on the *y*-axis. **(D)** The same experiment was performed using the human monoclonal IgE antibody 6D12. IC_50_ values based on each logistic fitted curve for WN subjects and 6D12 are shown in **(E)** All *R*^2^/Adjusted *R*^2^ values for each curve were reported as > 0.99 for subject 1 and > 0.85 for subject 2.

Competitive inhibition of IgE binding to JR2LS by individual and combinations of two WN VBPs at equimolar concentrations was assessed for IgE binding affinities and to determine if IgE with the same specificity recognizes the different VBPs (Figures 5A,B). nAH1.1 was not included because it was not bound by WNA sera. For this analysis, we assume that each VBP can accommodate binding by only one high-affinity IgE molecule at a time. The WNA sera displayed high but different binding affinities to the three WN VBPs, where one exhibited the highest IgE binding to JR2.3 (Figure 5A), while the other two sera tested here exhibited higher binding to JR2.1 and significantly lower binding for JR2.2 and JR2.3 (Figures 5B,C). It was expected that if combinations of competitor VBPs are binding to IgEs with different specificities an additive effect would occur. However, combinations of VBPs displayed logistic-fitted binding curves like those of individually preferred VBPs. For instance, the combination of JR2.2 and JR2.3 in Figure 5A competed similarly to JR2.3 alone. Or, in the case of combining JR2.1 with JR2.3, the inhibition curve resembles the JR2.1 curve more closely with reduced binding affinity than both JR2.1 and JR2.3, seemingly more competitive than additive. This same pattern was observed in the PW subject (Figure 5C), but with significantly higher binding affinity compared to the WN subjects, in which measuring the IC_50_ was well below the detection limit of our assay_._ Inhibition with VBP combinations was equal to or seemed to weaken the binding to the preferred VBP, likely due to dilution of the preferred binder.

When the monoclonal IgE antibody, 6D12, was used in the same experimental setup, the use of WN VBP combinations was not additive, indicating competition for binding to a monoclonal IgE. The binding of the monoclonal to the JR2LS, containing all three VBP domains, was not three times stronger. This indicated that only one of the VBPs can bind to the antibody at a time (Figure 5D), which was also supported by similar IC_50_ values for the corresponding curves in A and B (Figure 5E).

### 3.4 Linear and conformational IgE binding to VBPs differ between WNA, PNA, and PWA and non-allergic subjects

**Figure 6:**
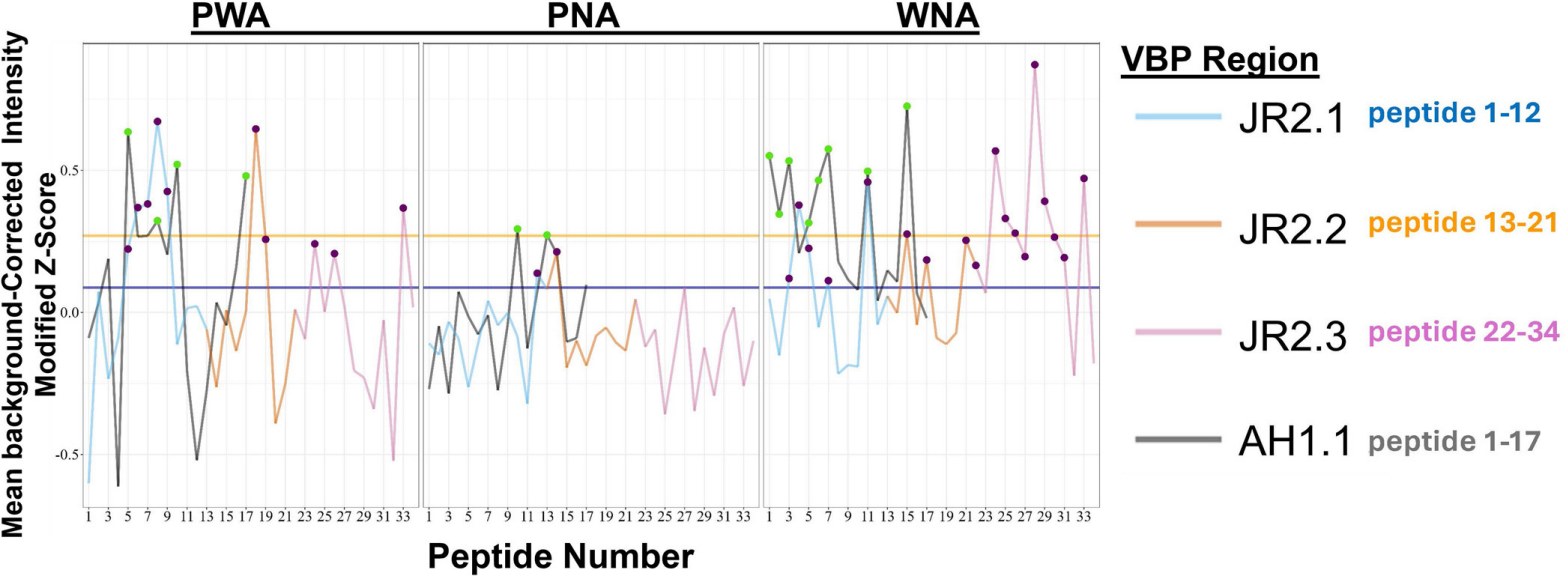
Modified z-scores for linear peptide IgE binding within VBP regions. Modified z-scores of background-corrected and normalized IgE binding intensity were calculated for each peptide within nAH1.1 and JR2LS VBPs (*x*-axis) and allergic status (panels), with mean modified z-scores (*y*-axis) calculated within each VBP per allergic status. Lines indicate binding for nAH1.1 and JR2LS, with the JR2.1, 2.2, and 2.3 segments colored to correspond with the peptides of each VBP. Orange and purple horizontal lines indicate the third quartiles for nAH1.1 and JR2LS, respectively. Peptides marked with dots indicate those with modified z-scores greater than the third quartile for nAH1.1 or JR2LS.

**Figure 7:**
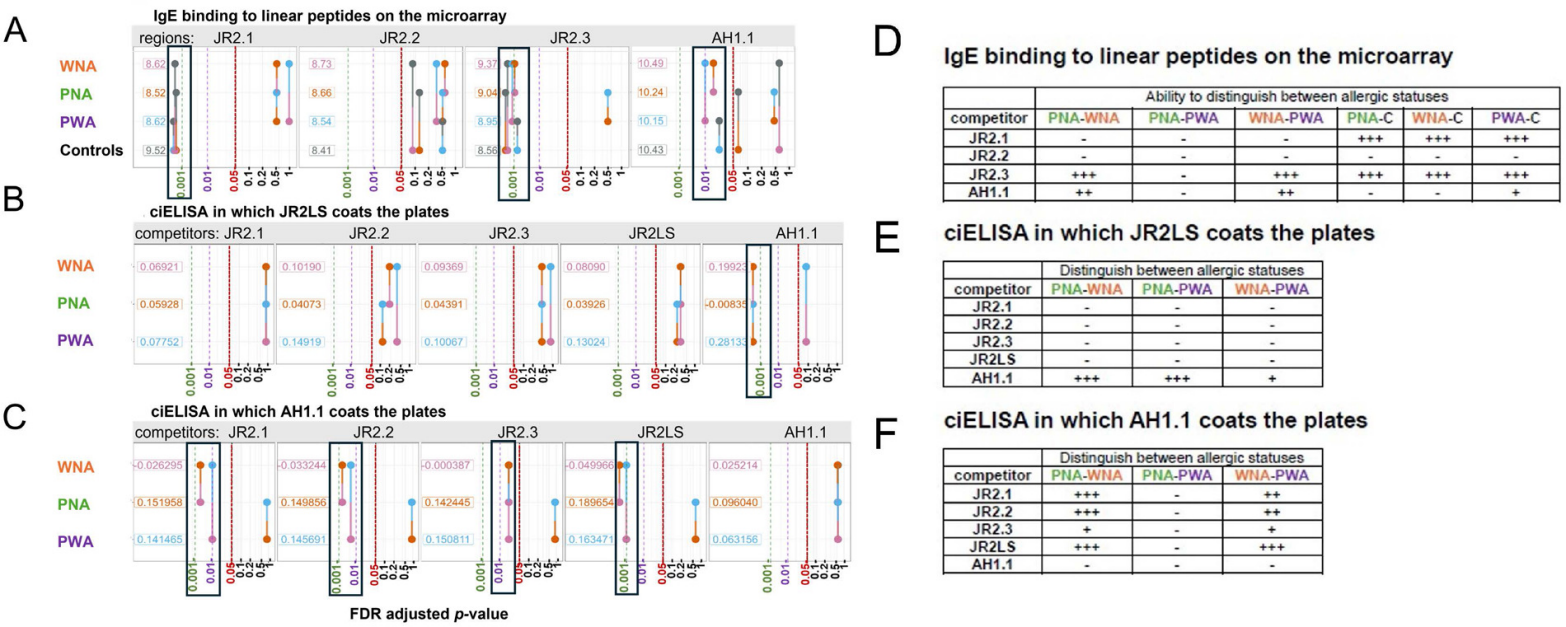
Ability to distinguish between allergic status using EMMs for linear and conformational peptides. EMMs of a fitted LMM for peptide microarray IgE binding intensity to linear peptide regions corresponding to ciELISA coats and targets were compared between allergic subject pools and nonallergic controls. The non-atopic controls were siblings to PN allergic subjects and confirmed nonallergic to both PN and WN. Pairwise comparisons of EMMs from a fitted LMM for ciELISA absorbance values (OD450) of conformational JR2LS **(C)** and nAH1.1 **(D)** VBP forms. The title of each panel in **(B)** indicates a linear peptide region, while the panel titles of **(C)** and **(D)** indicate the ciELISA Coat and binding Target combination (CxT). Colored values along the *y*-axis indicate EMMs for each allergic status group. Each comparison is associated with a vertical line segment colored in accordance with the EMMs of the groups being compared, and whose horizontal position is determined by the *p*-value of the comparison. False discovery rate (FDR)-adjusted *p*-values (*x*-axis) were calculated for EMM comparison *p*-values within each VBP, with adjusted *p*-values < 0.05 considered significant. From right to left, vertical dashed lines indicate *p*-value cutoffs of 0.05 (black), 0.01 (pink), and 0.001 (green). **D–F** shows summarized *p*-value significance values based on A-C. EMMs of a fitted LMM for peptide microarray IgE binding intensity to linear peptide regions of the VBPs were compared between allergic subject pools and nonallergic controls **(A)** Pairwise comparisons of EMMs from a fitted LMM for ciELISA absorbance values (OD_450_) of conformational JR2LS **(B)** and nAH1.1 **(C)** VBP forms were compared. A “+” designation indicates that that the VBP region (either linear or conformational) can distinguish between the two allergic status compared (PNA, WNA, or PWA) or the control (“C”).+indicates a *p*-value 0.01–0.05, ++ for 0.001–0.01, and +++ for <0.001. A “–”designation indicates a *p*-value >0.05, or not significant.

Synthetic overlapping 15 amino acid peptides (peptide number indicated on *x*-axis), representing the complete sequence of the four VBPs, were printed onto microarrays and assessed for IgE binding using sera from 30 total WNA, PNA, and PWA subjects (Figure 6; Table 1). Aligned with a previous study (14), similar IgE binding peptides were identified in the three subject groups within all four VBPs. For PNA subjects, the distinguishing peptides were in AH1.1 (A10, A13), JR2.1 (J12), and JR2.2 (J14). Interestingly, ten peptides were identified as significant in JR2.3 in the WNA group. These peptides in JR2.3 could be potential diagnostic indicators for a WN only allergy. Linear mixed effects models (LMMs) were generated for ciELISA and peptide microarray data using the lme4 package v1.1-34 in R to compare estimated marginal means (EMMs) for IgE binding to the VBP regions between allergic groups (Figure 7). EMMs are the predicted values of a dependent variable adjusted for the effects of other variables in the LMM and provide fairer and more reliable comparisons between groups, particularly with smaller sample sizes, vs. simple group means.

The linear and conformational VBP epitopes that can distinguish allergic status (WNA, PNA, PWA) are outlined Figures 7A–C, with trends summarized in Figures 7D–F. Significant differences in IgE binding intensity to a spanning peptide region (Figure 7A), in which all the peptides within a VBP were considered one region, are determined by an adjusted *p* < 0.05 (*x*-axis, values to the left of vertical red line) and summarized in Figure 7D. As an example, in Figure 7A, the colored circles connected by lines (representing allergic status on *y*-axis) within the black box show that the overall IgE binding to peptides of AH1.1 can distinguish between WNA and PWA (vertically connected circles at *p-*value = 0.01), between WNA and PNA, and PWA and controls (*p-*values between 0.01 and 0.05). The color in each circle indicates the predicted EMMs (numbers adjacent to each circle). Any circle (connected by lines) above a *p-*value of 0.05 (to the right of the dotted line) are not considered significant predictors of allergic status. In this same figure, linear epitope analysis shows significant differences for control (no nut allergies) vs. the allergic groups for AH1.1 (PWA), JR2.1, and JR2.3 (WNA, PNA, and PWA) (Figures 7A,D). IgE binding to linear JR2.2 failed to differentiate any groups.

The ciELISA OD_450_ values from Figure 3 and Figure 4 were used to determine significant differences in competitive IgE binding for each intact VBP for both plate coatings JR2LS (Figures 7B,E) and nAH1.1 (Figures 7C,F) between the three groups. For JR2LS coated plates, significant differences in IgE binding for WNA vs. PNA and PNA vs. PWA were observed for nAH1.1 competitor. No significant differences were observed between groups for nAH1.1 coated plates with nAH1.1 as a competitor. Significantly different EMMs were observed with JR2.1, JR2.2, JR2.3, and intact JR2LS as competitors for WNA vs. PNA and WNA vs. PWA but absent for PNA vs. PWA. These binding patterns can distinguish between different allergic cohorts.

## 4 Discussion

Plants have developed a variety of genomic defense mechanisms during growth and maturation, including the production of antimicrobial peptides (AMPs) known to have low primary sequence similarity. The n-terminal region of vicilins contains one or more VBPs that are most likely AMPs and share a common α-hairpinin structural motif, containing structure-stabilizing disulfide bonds (23, 24). These evolutionary conserved structures with variable amino acid sequences represent an example of cross-reactive IgE binding epitopes among species such as peanut and walnut. VBPs evolve and presumably duplicate over time to protect against evolving predators. While the amino acid sequence within each duplication changes to adapt, the conserved cysteine and helix-loop-helix or hairpinin motif are maintained throughout evolution, providing a naturally occurring, allergenic structural motif with embedded mutations and variable IgE binding patterns, which provides the ideal tools for assessing allergic cross-reactivity. This study investigates the possibility of diagnosing various allergic phenotypes by using linear and conformational IgE responses to this newly discovered class of plant seed allergens present in all vicilin-containing seeds. It is also likely that helix-loop-helix motifs found in dissimilar allergens and allergen families may contribute to clinically-relevant or irrelevant IgE cross-reactivity.

Previous studies utilized bioinformatic tools such as SDAP to predict potential IgE-reactive epitopes in similar peptide sequences using the property-distance function (25). With this method, a known immunodominant epitope of Ara h 2 was used to identify a cross-reactive, linear epitope in the JR2LS (16). The IgE binding regions identified in this study overlap or are near the previously described epitope containing areas, emphasizing the relevance of JR2LS VBPs as clinically relevant IgE targets (16, 17). Here, ELISA, linear peptide microarray, and computational analysis were used to explore the mechanism of cross-reactivity and diagnostic potential of IgE binding to conformational and linear peptides of PN and WN VBPs. The polyclonal nature of IgE in allergic disease complicates assessing its avidity and affinity, or the preference of a monoclonal IgE within sera for linear or conformational target epitopes. For example, a major conformational IgE epitope was identified for intact Ara h 2 using serum IgE binding to rAra h 2 and systematic replacement by rAra h 6 segments (17, 26). Studies on individuals with sustained unresponsiveness identified tolerance-associated conformational epitopes overlapping with known linear epitopes of Ara h 2, suggesting their importance in neutralizing IgE binding (27). IgE binding epitopes can also be assessed using monoclonal human IgE antibodies such as 6D12, which has specificity for JR2.1 and binds predominantly to a single site in its conformational form (Figure 1B). This led us to assume that each folded and intact VBP is capable of binding one IgE molecule at a given time. Previous work identified cross-reactive IgE linear epitopes between vicilin Jug r 2 and 2S albumin Ara h 2, including one within JR2.1 peptide 5 (the primary target of 6D12) on the linear microarray (16). It was generally hypothesized that for cross-reactivity to occur, a sequence similarity threshold of approximately 35% across 80 amino acids existed between the IgE epitopes (28). However, VBPs in both WN and PN were found to be cross-reactive due to their common α-hairpinin motif regardless of low sequence identity (14). For Jug r 2 and Ara h 2, cross-reactivity can still be mediated at the linear epitope level despite below-threshold sequence similarity (16, 17). A custom anti-JR2LS antibody against folded Jug r 2 detected PN and related tree nut allergens in ELISA and Western Blot, where reactive bands confirmed as known allergens by mass spectrometry (17).

In the current study, ciELISAs showed IgE binding differences to the conformational epitopes most likely due to sequence diversity among the VBPs. When competing for IgE binding to intact JR2LS, nAH1.1 competed the least in WNA and PWA sera and exhibited the lowest IgE binding among the VBPs (Supplementary Figure S1) with a maximum potential inhibition below 50%. Consistent with a previous study (14), the folded JR2.1 was the most frequent highest competitive inhibitor for binding to the intact JR2LS, and 17 out of 20 subjects showed JR2.1 inhibiting IgE binding to JR2LS by ≥50% at one or both high competitor concentrations (Supplementary Figure S1). Interestingly, 16 out of 20 subjects exhibited ≥50% binding inhibition to JR2LS with either JR2.2 or JR2.3, including with sera from a PNA subject. The maximum level of inhibition was similar in majority of the subjects in all groups for JR2.2 and JR2.3. In contrast to nAH1.1 coated plates, competitive inhibition of IgE binding to individual WN VBPs and JR2LS dropped considerably with all subject groups frequently under 50% inhibition (Supplementary Figure S2). nAH1.1 was the top competitor among the PNA group (Supplementary Figure S2B), which makes it an advantageous candidate for inclusion in a potential diagnostic panel. We concluded that although linear IgE epitopes predominantly reside in JR2.1, conformational epitopes within other VBPs are IgE-reactive in all three subject groups. It appears a combination of (perhaps overlapping) sequence and structure determines cross-reactivity to more than one allergen.

The next objective was to determine whether structurally similar VBPs were recognized by IgE from a single B cell with varying affinities due to distinct amino acid sequences or by IgE from multiple B cells with subtle specificity differences. Individual sera with IgE binding preferences for different VBPs were selected to gain an understanding of the IgE affinity and specificity. Based on competitive IgE binding, the immunodominant VBP fragment(s) differed between the WNA subjects (i.e., JR2.3 for Subject 1; JR2.1 for Subject 2) and PWA (JR2.1 for Subject 30). In cases where partial inhibition is seen by some VBPs for certain sera, this may reflect overlapping linear and conformational epitopes which supports the notion that IgE binding to VBPs is influenced by both sequence variability and stable hairpin structures. Though JR2.1 contained the immunodominant linear epitopes, conformational binding determined that all three WN VBPs should be considered as relevant for IgE binding and cross-reactivity. Combining equimolar levels of WN VBPs did not reveal a significant additive IgE-binding effect, indicating competition for IgE molecules with the same specificity in subject sera, most likely from the same B cell clone. This was consistent when WN subject sera showed very little difference in binding curves between preferential individual VBPs and their corresponding combinations. Competitive binding using human IgE monoclonal antibody 6D12 further supported this finding. Notably, the intact JR2LS may fold to accommodate only one IgE molecule with its preferred VBP, possibly due to steric hindrance.

Statistical calculations performed on this small group of subjects demonstrate that IgE binding to linear and conformational epitopes of these small, yet ubiquitous allergens allow us to distinguish allergic phenotypes. Based on z-scores of linear peptide IgE binding, PNA sera recognized two immunodominant epitopes in AH1.1 with only one epitope in JR2.1 and JR2.2. However, both WNA and PWA sera bind a higher number of linear immunodominant epitopes in all four VBPs. Already, the distinction between having or not having a WN allergy based on the linear IgE epitopes is evident. This is also supported by our statistical modeling of these subjects against nonallergic controls. We used LMM to evaluate our data, which we believe is best suited to assessing small data sets often inevitable in clinical studies. The LMM of ciELISA data reveals conformational IgE binding patterns to VBPs based on allergic status. For example, when competing for sera IgE binding against nAH1.1, all three individual WN VBPs had significant binding differences between WNA vs. PNA and WNA vs. PWA subjects. The analysis of conformational and linear IgE epitopes of VBPs as a simple model system highlights these fragments' potential to help discriminate WNA, PNA, and PWA. Further studies with larger numbers and blind samples to validate the prediction potential of this model are planned. The presence of the highly conserved IgE-binding conformational motif among these VBPs and similar hairpin-like structures in other allergens (such as Ara h 2) may assist in identifying the structures that contribute most to clinically-relevant cross-reactivity. Integrating data on both types of epitopes in future predictive models could enhance diagnostic accuracy.

In consideration of our findings, it is important to acknowledge several limitations that may impact the interpretation and generalizability of our study. First, our sample selection may be biased, as the ciELISA and microarray experiments were conducted exclusively on sera that exhibited detectable IgE binding to VBPs. This focus potentially restricts the applicability of our results to a wider allergic population. Additionally, the relatively small sample size of 30 subjects included in our detailed analyses may limit the statistical power of our findings and adversely affect the reliability of subgroup comparisons. Furthermore, we did not account for potential confounding factors such as age, co-allergies, and total serum IgE levels, all of which could influence IgE binding patterns and contribute to variability in the data. Finally, while the use of the monoclonal IgE antibody (6D12) has provided us with valuable mechanistic insights, it may not fully capture the complexity and diversity of polyclonal IgE responses that are typically observed in human sera. Addressing these limitations in future research will be crucial for enhancing our understanding of cross-reactivity among nut allergens and improving the utility of VBPs as diagnostic tools in food allergy.

## Data Availability

The original contributions presented in the study are included in the Supplementary Material, and further inquiries can be directed to the corresponding author.

## References

[1] Jones SM Burks AW. Food allergy. N Engl J Med. (2017) 377(23):2294–5. 10.1056/NEJMcp1611971 29211665

[2] Warren C Lei D Sicherer S Schleimer R Gupta R. Prevalence and characteristics of peanut allergy in US adults. J Allergy Clin Immunol. (2021) 147(6):2263–70.e5. 10.1016/j.jaci.2020.11.046 33579526 PMC12341317

[3] Kamath SD Bublin M Kitamura K Matsui T Ito K Lopata AL. Cross-reactive epitopes and their role in food allergy. J Allergy Clin Immunol. (2023) 151(5):1178–90. 10.1016/j.jaci.2022.12.827 36932025

[4] Chruszcz M Kapingidza AB Dolamore C Kowal K. A robust method for the estimation and visualization of IgE cross-reactivity likelihood between allergens belonging to the same protein family. PLoS One. (2018) 13(11):e0208276. 10.1371/journal.pone.0208276 30496313 PMC6264518

[5] Aalberse RC. Structural biology of allergens. J Allergy Clin Immunol. (2000) 106(2):228–38. 10.1067/mai.2000.108434 10932064

[6] Aalberse RC. Assessment of allergen cross-reactivity. Clin Mol Allergy. (2007) 5:2. 10.1186/1476-7961-5-2 17291349 PMC1797810

[7] Dramburg S Hilger C Santos AF de Las Vecillas L Aalberse RC Acevedo N EAACI molecular allergology user’s guide 2.0. Pediatr Allergy Immunol. (2023) 34(Suppl 28):e13854. 10.1111/pai.13854 37186333

[8] Radauer C Breiteneder H. Evolutionary biology of plant food allergens. J Allergy Clin Immunol. (2007) 120(3):518–25. 10.1016/j.jaci.2007.07.024 17689599

[9] Koppelman SJ Hefle SL Taylor SL De Jong GA. Digestion of peanut allergens Ara h 1, Ara h 2, Ara h 3, and Ara h 6: a comparative *in vitro* study and partial characterization of digestion-resistant peptides. Mol Nutr Food Res. (2010) 54(12):1711–21. 10.1002/mnfr.201000011 20603832

[10] Zhang J Payne CD Pouvreau B Schaefer H Fisher MF Taylor NL An ancient peptide family buried within vicilin precursors. ACS Chem Biol. (2019) 14(5):979–93. 10.1021/acschembio.9b00167 30973714

[11] Burks AW Williams LW Helm RM Connaughton C Cockrell G O'Brien T. Identification of a major peanut allergen, Ara h I, in patients with atopic dermatitis and positive peanut challenges. J Allergy Clin Immunol. (1991) 88(2):172–9. 10.1016/0091-6749(91)90325-I 1880317

[12] Aalberse RC Mueller GA Derksen NIL Aalberse JA Edwards LL Pomes A Identification of the amino-terminal fragment of Ara h 1 as a major target of the IgE-binding activity in the basic peanut protein fraction. Clin Exp Allergy. (2020) 50(3):401–5. 10.1111/cea.13554 31880850 PMC7047623

[13] Maleki SJ Kopper RA Shin DS Park C-W Compadre CM Sampson H Structure of the major peanut allergen Ara h 1 may protect IgE-binding epitopes from degradation. J Immunol. (2000) 164(11):5844–9. 10.4049/jimmunol.164.11.5844 10820263

[14] Foo ACY Nesbit JB Gipson SAY Cheng H Bushel P DeRose EF Structure, immunogenicity, and IgE cross-reactivity among walnut and peanut vicilin-buried peptides. J Agric Food Chem. (2022) 70(7):2389–400. 10.1021/acs.jafc.1c07225 35139305 PMC8959100

[15] Foo ACY Nesbit JB Gipson SAY DeRose EF Cheng H Hurlburt BK Structure and IgE cross-reactivity among cashew, pistachio, walnut, and peanut vicilin-buried peptides. J Agric Food Chem. (2023) 71(6):2990–8. 10.1021/acs.jafc.2c07061 36728846 PMC10402694

[16] Maleki SJ Teuber SS Cheng H Chen D Comstock SS Ruan S Computationally predicted IgE epitopes of walnut allergens contribute to cross-reactivity with peanuts. Allergy. (2011) 66(12):1522–9. 10.1111/j.1398-9995.2011.02692.x 21883278 PMC3203311

[17] Nesbit JB Schein CH Braun BA Gipson SAY Cheng H Hurlburt BK Epitopes with similar physicochemical properties contribute to cross reactivity between peanut and tree nuts. Mol Immunol. (2020) 122:223–31. 10.1016/j.molimm.2020.03.017 32442779

[18] Downs ML Semic-Jusufagic A Simpson A Bartra J Fernandez-Rivas M Rigby NM Characterization of low molecular weight allergens from English walnut (Juglans regia). J Agric Food Chem. (2014) 62(48):11767–75. 10.1021/jf504672m 25388987

[19] Wurth MA Hadadianpour A Horvath DJ Daniel J Bogdan O Goleniewska K Human IgE mAbs define variability in commercial Aspergillus extract allergen composition. JCI Insight. (2018) 3(20):e123387. 10.1172/jci.insight.123387 30333320 PMC6237483

[20] Rambo IM Kronfel CM Rivers AR Swientoniewski LT McBride JK Cheng H IgE and IgG4 epitopes of the peanut allergens shift following oral immunotherapy. Front Allergy. (2023) 4:1279290. 10.3389/falgy.2023.1279290 38093814 PMC10717846

[21] Kronfel CM Cheng H McBride JK Nesbit JB Krouse R Burns P IgE epitopes of Ara h 9, Jug r 3, and Pru p 3 in peanut-allergic individuals from Spain and the US. Front Allergy. (2022) 3:1090114. 10.3389/falgy.2022.1090114 36698378 PMC9869384

[22] Burks AW Shin D Cockrell G Stanley JS Helm RM Bannon GA. Mapping and mutational analysis of the IgE-binding epitopes on Ara h 1, a legume vicilin protein and a major allergen in peanut hypersensitivity. Eur J Biochem. (1997) 245(2):334–9. 10.1111/j.1432-1033.1997.t01-1-00334.x 9151961

[23] Bakare OO Gokul A Fadaka AO Wu R Niekerk LA Barker AM Plant antimicrobial peptides (PAMPs): features, applications, production, expression, and challenges. Molecules. (2022) 27(12):3703. 10.3390/molecules27123703 35744828 PMC9229691

[24] Ozias-Akins P Breiteneder H. The functional biology of peanut allergens and possible links to their allergenicity. Allergy. (2019) 74(5):888–98. 10.1111/all.13719 30636003 PMC6563476

[25] Ivanciuc O Midoro-Horiuti T Schein CH Xie L Hillman GR Goldblum RM The property distance index PD predicts peptides that cross-react with IgE antibodies. Mol Immunol. (2009) 46(5):873–83. 10.1016/j.molimm.2008.09.004 18950868 PMC2651743

[26] Chen X Negi SS Liao S Gao V Braun W Dreskin SC. Conformational IgE epitopes of peanut allergens Ara h 2 and Ara h 6. Clinical & Experimental Allergy. (2016) 46(8):1120–8. 10.1111/cea.12764 27238146 PMC4963300

[27] LaHood NA Min J Keswani T Richardson CM Amoako K Zhou J Immunotherapy-induced neutralizing antibodies disrupt allergen binding and sustain allergen tolerance in peanut allergy. J Clin Invest. (2023) 133(2):e164501. 10.1172/JCI164501 36647835 PMC9843057

[28] McClain S. Bioinformatic screening and detection of allergen cross-reactive IgE-binding epitopes. Mol Nutr Food Res. (2017) 61(8):1600676. 10.1002/mnfr.201600676 28191711 PMC5573986

