# Supplementary figures and images for: Linear and conformational epitopes of vicilin-buried peptides as a model for improved nut allergy diagnostics
Source: Front Allergy. 2025 Sep 22;6:1648262. doi: 10.3389/falgy.2025.1648262 (PMC12497738; doi:10.3389/falgy.2025.1648262)

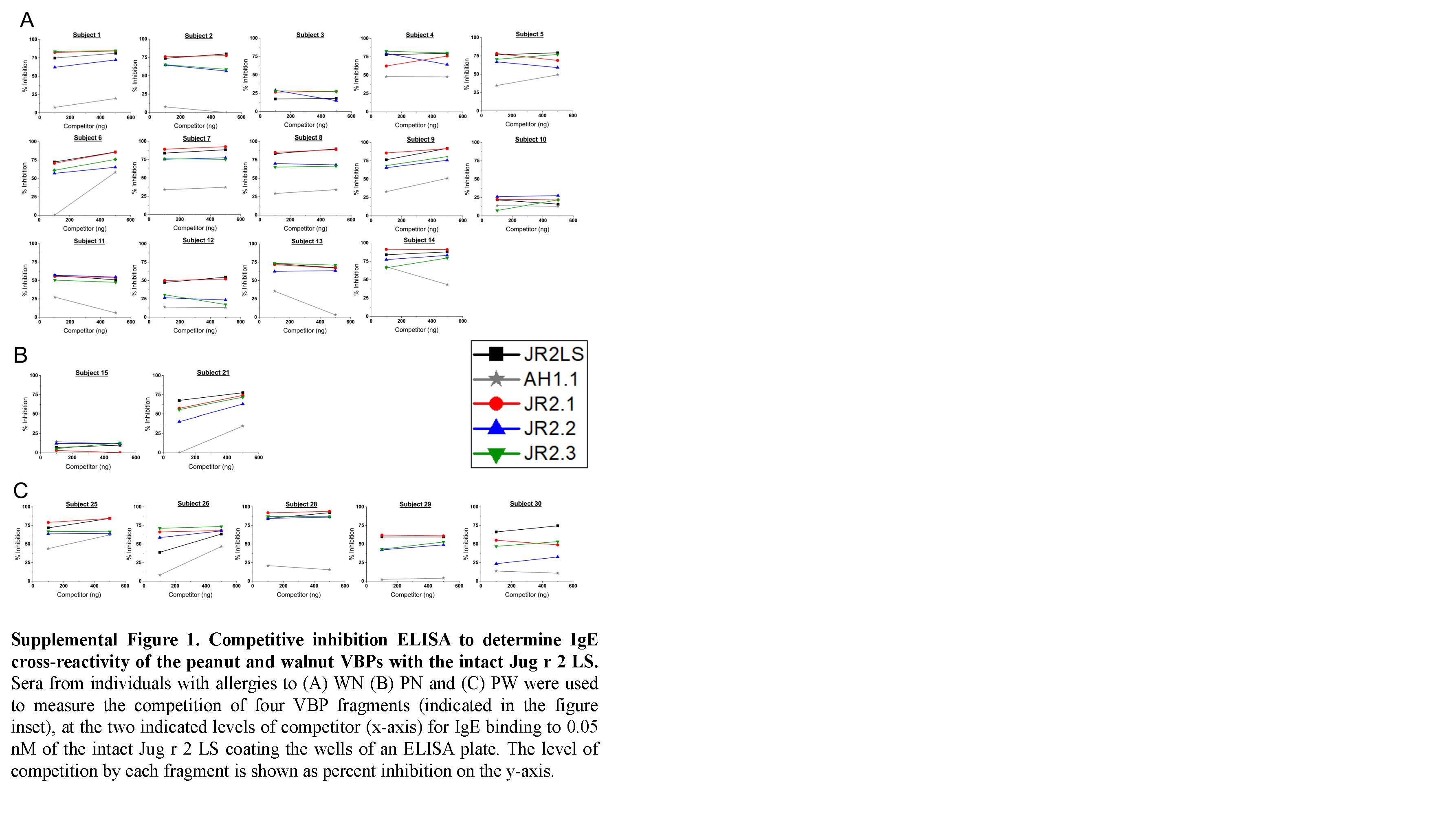

Supplement: Supplementary file 1 [file Image1.jpeg]

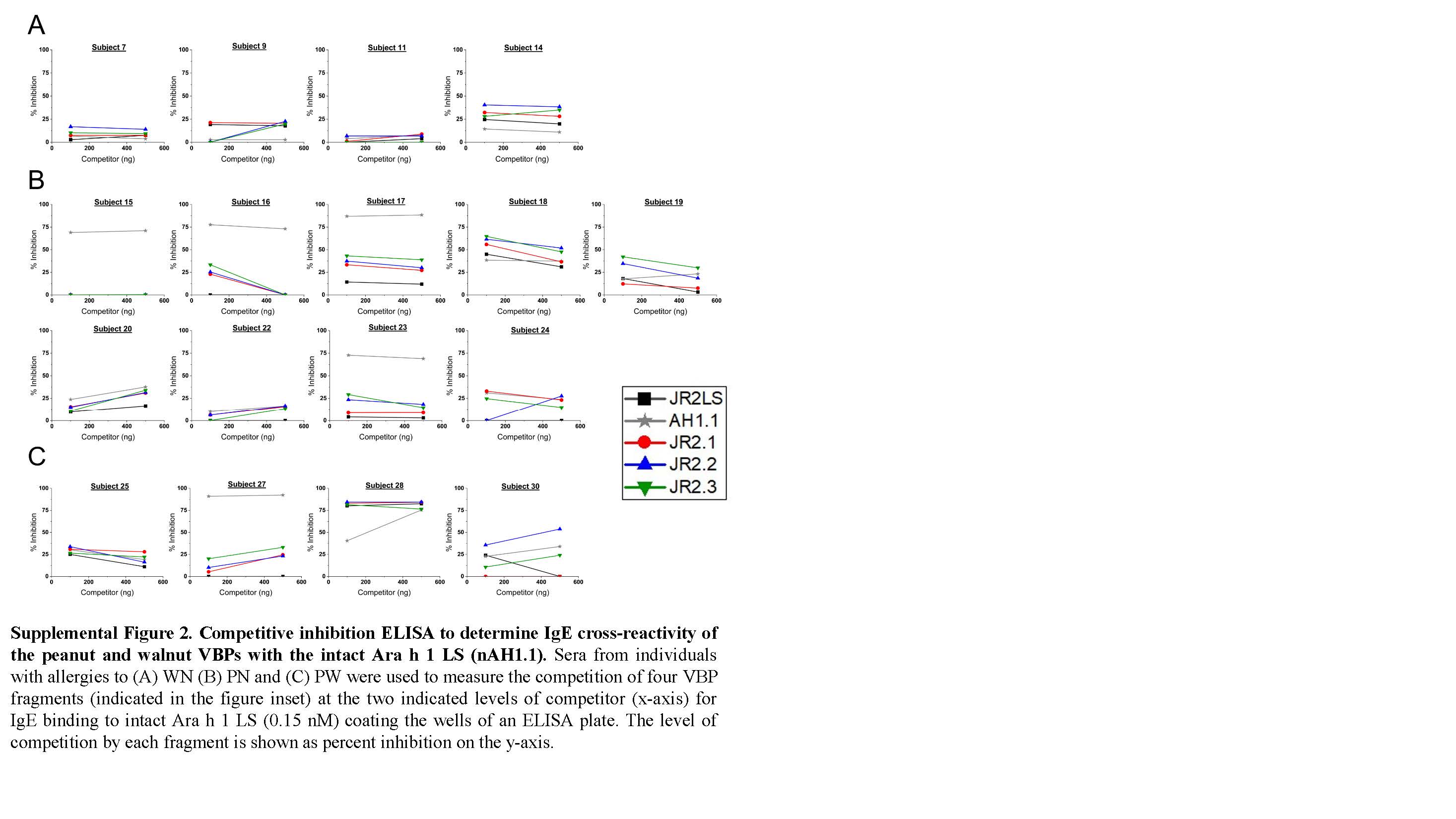

Supplement: Supplementary file 2 [file Image2.jpeg]
