# Supplementary material for: Linear and conformational epitopes of vicilin-buried peptides as a model for improved nut allergy diagnostics
Source: Front Allergy. 2025 Sep 22;6:1648262. doi: 10.3389/falgy.2025.1648262 (PMC12497738; doi:10.3389/falgy.2025.1648262)
Supplement: Supplementary file 4 [file Table1.docx]

Supplementary Material

# Supplementary Data

Below describes detailed methods on the statistical analyses performed in this study. Peptide microarray data were processed as previously described (1). In brief, GenePix GPR output files containing IgE signal intensities were processed with the Limma package v3.52.4 using R v4.2.1 and RStudio Build 548 v2022.07.0 (34ea3031, 2022-07-06) for Windows. Median pixel intensities at 532 nm (IgE) were background corrected using backgroundCorrect.matrix function in Limma (offset = 10, method = “normexp”), and the background-corrected values were base 2 logarithm transformed. Peptides that were not present in all arrays and arrays completely missing required peptides were removed from analysis. Cyclic-Loess normalization for each peptides was performed between all possible pairs of arrays for 3 iterations, with a loess smoothing window span of 0.3 using the normalizeBetweenArrays function in Limma (method = “cyclicloess”, cyclic.method = “pairs”).

For ciELISA, mean OD_450_ values were obtained per individual (PN allergic = 9; WN allergic = 14; WN/PN allergic = 5) for each coat-binding target combination, and the overall distribution was assessed for skewness and kurtosis using the skewness() and kurtosis() functions in the moments package v0.14.1. Mean OD_450_ values were transformed to a more Gaussian distribution using the Gaussianize() function for skewed data in LambertW v0.6.9-1 package (parameters: method = “IGMM”, type = “s”). For peptide microarray data (PN allergic = 22; WN allergic = 12; WN/PN allergic = 12, non-atopic siblings of peanut allergic individuals (Control) = 10), peptides corresponding to walnut VBPs and nAH1.1 used in ciELISA were selected, and the background corrected and normalized log2 intensity values within each region were transformed to be more Gaussian using Gaussianize() function for skewed data in LambertW v0.6.9-1 package (parameters: method = “IGMM”, type = “s”).

LMMs were generated for ciELISA and linear peptide regions in R using the lme4 v1.1-34 package (2). The ciELISA LMM focused on OD_450_ (IgE binding) as the dependent variable and was structured to investigate the effects of ELISA coat (C) and binding target (T) combinations (CxT) along with their interaction with allergic status, accounting for potential clustering within patients. The model formulation is as follows: *OD_450_ ~ CxT * allergic status + (1 | participant id)*. The model allows for estimation of fixed effects associated with interactions among ciELISA coat/binding target and allergic status, while accommodating for random intercepts for individual participants.

The LMM used for peptide microarray data is detailed as followed. The model was specified as follows: *IgE intensity ~ VBP region * allergic status + (1 | participant id) + (1 | peptide number:VBP region)*. The model formulation allows for estimation of fixed effects associated with the interaction between allergic status and VBP region, while accommodating random intercepts for individual participants and the interaction between peptide number and the VBP region. The inclusion of random effects enables the model to capture variability arising from repeated measures within patients across different peptides within VBP regions. Performance of each model was assessed using the check_model() function in performance v0.10.4 (QQ-plot, normality, linearity, homogeneity of variance, outliers, QQ-plot for random effects, and posterior predictive checks). EMMs were computed using the emmeans() function in emmeans v1.8.7 (3), and pairwise comparisons of the treatment means was performed using the contrast() function in emmeans v1.8.7 (parameters: methods = “pairwise”). False discovery rate control of comparison *p*-values was performed via Benjamini-Hochburg (FDR) correction using p.adjust()(method = “fdr”) in the stats package. Pairwise *p*-value plots of between-group comparisons were generated with the pwpp() function in emmeans v1.8.7 (3) and customized with ggplot2 v3.4.2 (4).

1. Rambo IM, Kronfel CM, Rivers AR, Swientoniewski LT, McBride JK, Cheng H, et al. IgE and IgG4 epitopes of the peanut allergens shift following oral immunotherapy. Front Allergy. 2023;4:1279290.

2. Bates D, Mächler M, Bolker BM, Walker SC. Fitting Linear Mixed-Effects Models Using lme4. J Stat Softw. 2015;67(1):1-48.

3. Lenth RV. emmeans: Estimated Marginal Means, aka Least-Squares Means. 1.8.7 ed2023.

4. Wickham H. Elegant Graphics for Data Analysis. New York: Springer-Verlag; 2016.
